# Parent-adolescent intergenerational transmission of distress: the roles of family climate, adolescents’ self-compassion and mindfulness

**DOI:** 10.3389/fpsyg.2025.1595515

**Published:** 2025-08-14

**Authors:** Shuying Zhou, Chi Kin Kwan, Yueyue Ai, Ying Ma

**Affiliations:** ^1^Shaanxi Normal University, Xi’an, China; ^2^Department of Social and Behavioural Sciences, City University of Hong Kong, Hong Kong SAR, China; ^3^Xi’an Jiaotong University Kindergarten, Xi’an, China

**Keywords:** psychological distress, family emotional climate, mindfulness, self-compassion, adolescence

## Abstract

**Background:**

Existing research indicates that parental psychological distress correlates with adverse psychological outcomes in adolescents. Nevertheless, limited studies have delved into the mechanisms underpinning this relationship.

**Objectives:**

This study aimed to examine whether the link between parental psychological distress and adolescent psychological distress is mediated by the family emotional climate, as well as adolescents’ mindfulness and self-compassion.

**Methods:**

The study sample consisted of 348 parent-adolescent dyads. Adolescents (57.5% female) had a mean age of 14.21 years (SD = 1.74, age range: 12–18 years). Parents reported their psychological distress, while adolescents completed measures of family emotional climate, mindfulness, self-compassion, and psychological distress.

**Results:**

The results indicated that parents’ psychological distress was indirectly linked to adolescents’ psychological distress via the family emotional climate, adolescent mindfulness, and self-compassion. Furthermore, the path models were found to be invariant across different age stages and sexes of adolescents.

**Conclusion:**

Mindfulness and self-compassion appear to develop within a family emotional environment marked by care and harmony. These two personal resources, in conjunction with parents’ psychological distress, are associated with adolescents’ psychological distress.

## Introduction

Adolescence, marked by rapid physical, psychological, personality, and social changes, is a developmental stage that heightens adolescents’ vulnerability to stressors. These ongoing stressors may contribute to the onset of mood disorders (Neppl et al., 2024). Crucially, adolescent emotional distress often extends into adulthood and is associated with higher rates of mood disorders—including affective and anxiety disorders—in later life (Lin et al., 2024; Neppl et al., 2024). Research indicates that family factors, such as parental warmth and rejection, parenting styles, and family environment, play a pivotal role in shaping adolescents’ mental health outcomes (Sun et al., 2022; Hammen and Brennan, 2002). Early life experiences exert lasting influences on health and behavioral patterns across the lifespan, with effects potentially persisting across generations (Park, 2020). This intergenerational continuity is conceptualized as “intergenerational transmission,” a process wherein parental exposures, traits and behavior—such as stress responses, mental health vulnerabilities, parenting—profoundly impact offspring development (Bowers and Yehuda, 2016; Park, 2020; Rothenberg et al., 2023). However, the mechanisms underlying the effect of parental psychological distress on adolescent psychological distress through intergenerational transmission deserves further explore.

### Intergenerational transmission of psychological distress

Psychological distress encompasses symptoms of anxiety, depression, and stress (Cummings et al., 2014). Research consistently underscores the critical influence of parental psychological states on adolescent psychological distress (Park, 2020; Neppl et al., 2024). Stressed parents may confer vulnerability through genetic risk factors or behavioral changes resulting from stress-related psychopathology (Bowers and Yehuda, 2016). For instance, studies demonstrate that depression and anxiety often aggregate within families Children of parents with obsessive-compulsive disorder, anxiety, or depression are more likely to experience negative outcomes such as poor school performance, depression, and oppositional defiant disorder (Neppl et al., 2024; Sawyer et al., 2019). This heightened risk is frequently attributed to intergenerational transmission—a process whereby parental stress or psychopathology impacts parental psychology and behavior, induces biological changes, and ultimately affects offspring outcomes (Bowers and Yehuda, 2016). Supporting this view, a meta-analysis grounded in interpersonal acceptance–rejection theory (IPARTheory) confirms that humans fundamentally require positive responses from close attachment figures (Khaleque and Ali, 2017). Consequently, exposure to parental negative emotions significantly increases children’s likelihood of experiencing diverse adverse mental health outcomes, potentially persisting into adulthood. Specific mechanisms underlying this transmission include the tendency of psychologically distressed parents to have a lower threshold for aversive experiences. This heightened sensitivity promotes the retrieval of negative memories, predisposing parents to negatively evaluate their children and exhibit poor parenting behaviors (e.g., inconsistent discipline, poor monitoring). Adolescents may then internalize these negative interactions, developing core feelings of being “unlovable” or “incapable,” which form a foundation for mental health issues (Sun et al., 2022). Research has established that parental psychological and social characteristics can influence children through intergenerational transmission (Rothenberg et al., 2023; Yoshida and Busby, 2012). Various parental behaviors, such as eating habits, aggressive behavior, inconsistent discipline, and poor monitoring, can be transmitted to children (Kim et al., 2009; Rothenberg et al., 2023). In conclusion, intergenerational transmission represents a key pathway linking parental and adolescent psychological distress. One aim of this study was to investigate the direct effects of parental psychological distress on adolescent psychological distress through the lens of intergenerational transmission.

### Mediator of family emotional climate

As evidence of intergenerational transmission between parental psychological distress and adolescent psychological distress has increased, some studies have attempted to understand the mechanisms or mediators that may account for this relationship. For instance, Van Loon et al. (2013) found that parental psychological distress was associated with adolescents’ psychological distress through parent-child interaction (e.g., parental monitoring and parental support) and family environment (e.g., cohesion, expressiveness, and conflict). Roustit et al. (2010) indicated that adolescents’ self-esteem, parental emotional support, and extra-parental social support have been found to serve as mediators in the parent-adolescent transmission of psychological problems. In conclusion, although peer relationships become increasingly important during adolescence, the family remains one of the most important relationship systems during this period (Van Eickels et al., 2022). Thus, the impact of family emotional climate on adolescents may particularly significant due to its pervasive and diffuse nature in many family interactions (Ogbaselase et al., 2022). Within this context, parental warmth, acceptance, and support prove fundamental to adolescent emotional wellbeing (Lin et al., 2024), reinforcing the critical function of family environment in moderating intergenerational distress transmission.

Relatedly, the family emotional climate generally refers to the overall context of family members’ emotional states, expression, and regulation processes (Morris et al., 2007). This climate often manifests in two distinct forms. A positive climate characterized by warmth, support, acceptance, and trust, and a negative climate marked by interpersonal conflict, pervasive negative emotions, and maladaptive emotion regulation (Kurdek et al., 1995). Notably, parents experiencing psychological distress often lack the capacity to cultivate a positive emotional climate (Aktar and Bögels, 2017). As Hammen’s (2006) intergenerational interpersonal stress model posits, children of depressed parents in stressful environments face elevated depression risk. This occurs because depressed parents typically exhibit flattened or negative affect during interactions, creating suboptimal interpersonal environments (Aktar and Bögels, 2017). Similarly, parents with high levels of anxiety may have significantly worse interactions with their children due to negative emotions and may not be able to provide an optimal home environment (Liu et al., 2021). Such negative climates predispose adolescents to heightened emotional reactivity, insecurity, and internalizing symptoms like depression and anxiety (Stuart and Jose, 2012). Adolescents exposed to frequent negative family emotional climates, such as harsh parenting, anger, or family conflict, may experience greater maladjustment, including internalizing and externalizing problems, as well as emotional dysregulation (Modry-Mandell et al., 2006). Conversely, positive emotional climates foster greater adolescent happiness, adaptive emotion regulation, and reduced social dysregulation (Lin et al., 2024; Lai and McBride-Chang, 2001). Additionally, a supportive extended family can enhance adolescents’ psychological resources, reduce emotional distress, and help resolve difficulties (Lin and Guo, 2024). Given the family emotional climate’s pivotal mediating role between parental and adolescent distress, this study specifically investigates its indirect effects in transmitting psychological distress across generations.

### Mediators of adolescents’ self-compassion and mindfulness

It is essential to note that family practice is more often participated as an indirect factor affecting adolescent mental health development (Lin and Guo, 2024). For instance, a negative family atmosphere heightens adolescents’ risk for depression and anxiety by impairing the development of adaptive emotion regulation strategies (Van Eickels et al., 2022). Conversely, a positive family environment aids in cultivating the ability to use cognitive strategies to reinterpret negative events, which is crucial for reducing mental illness (Lin and Guo, 2024). Thus, adolescents’ emotion regulation strategies may mediate the relationship between family emotional climate and psychological distress. Research also suggests that self-compassion and mindfulness are individual dispositions that develop within the family environment (Caldwell and Shaver, 2015; Neff and McGehee, 2010), and which might help enhance emotion regulation and reduce psychological disorders (Yuan et al., 2023; Zhou et al., 2022).

Rooted in Buddhism, mindfulness is defined as “paying attention in a particular way: on purpose, in the present moment, non-judgmentally” (Kabat-Zinn, 1994, p. 4). Individuals exhibit varying levels of dispositional mindfulness, which is positively linked to wellbeing aspects such as positive emotions, emotional self-regulation, emotional intelligence, and life satisfaction (Brown and Ryan, 2003; Jimenez et al., 2010; Chen and Deng, 2022; Makadi and Koszycki, 2019). Conversely, mindfulness is negatively related to depression, anxiety, and stress (Chen and Deng, 2022; Jimenez et al., 2010). Highly mindful individuals are more likely to pause before reacting to negative emotions, disengage from automatic thoughts, and focus on the present moment (Bishop, 2004; Yuan et al., 2023). This metacognitive stance facilitates insight into emotional responses while promoting adaptive emotion regulation and rational problem-solving strategies-key protective factors for mental health (Gross, 2014; Preisig and Neuenschwander, 2024). Importantly, family emotional climate fosters these capacities through attachment security, with which it demonstrates significant correlation (Yuan et al., 2023). Securely attached adolescents typically approach distressing experiences with calm attentiveness rather than suppression or avoidance, owing to reduced abandonment fears and enhanced acceptance of negative affect (Ryan et al., 2007; Yuan et al., 2023). This may facilitate the development of higher mindfulness levels in adolescents. Conversely, research confirms that insecure attachment correlates with diminished mindfulness (Pepping et al., 2014). Research further emphasizes how family factors shape the neurobiology of emotion regulation (Lin et al., 2024). Consequently, family emotional climate emerges as a significant correlate of both adolescent psychological distress and mindfulness development.

Self-compassion, an attitude of treating oneself and others with kindness and understanding in the face of suffering (Neff, 2003; Moreira et al., 2018). It especially emphasizes self-kindness with oneself rather than overidentification or harsh self-judgment; and common humanity versus isolation, which emphasizes that failure and mistakes are universal for all humans rather than feelings of isolation and uniqueness (Neff, 2003). This disposition develops within secure relational contexts characterized by warmth and acceptance (Gilbert and Procter, 2006; Shaver et al., 2016). Positive family factors seem to contribute to adolescents’ self-compassion development, which in turn impacts their mental health. Neff and McGehee (2010) suggested that harmonious family functioning and secure attachment are positively associated with higher levels of self-compassion. Growing up in a positive family emotional climate, individuals feel connectedness and warmth, which enables them to relate to themselves in a caring and compassionate manner. When facing difficult circumstances, highly self-compassionate individuals are better able to comfort themselves, avoid over-judging their shortcomings, and maintain balance in their experiences (Zhou et al., 2022), which may reduce psychological maladjustment. Conversely, negative family climates marked by conflict and threat cultivate self-critical tendencies (Gilbert and Procter, 2006), which elevate vulnerability to psychological distress (Lin and Guo, 2024). Empirical evidence confirms self-compassion’s protective role, which correlates negatively with anxiety (Makadi and Koszycki, 2019) and positively with wellbeing (Neff and McGehee, 2010). Therefore, this study also aims to explore the indirect effects of family emotional climate on adolescent psychological distress through adolescents’ mindfulness and self-compassion.

### Age and sex differences in adolescents’ mindfulness and self-compassion

This study examines the role of mindfulness and self-compassion in the relationship between parental and adolescent psychological distress, with particular attention to developmental stage and sex differences. Evidence suggests that mindfulness and self-compassion may vary between different stages of adolescence and between sexes. For instance, adolescent boys tend to demonstrate higher mindfulness levels than girls (Moreira et al., 2018). Regarding self-compassion, Bluth and Blanton (2015) reported markedly lower levels among high school girls compared to boys. However, other studies, such as that by Muris et al. (2016), found no significant sex differences in self-compassion when analyzing a sample of adolescents aged 12–17 years as a whole. Similarly, Neff and McGehee (2010) found no significant differences between boys and girls in a sample of adolescents aged 14–17 years. Additionally, Pang et al. (2024) found that self-compassion negatively predicted depression 6 months later in boys, but not in girls. These studies underscore the importance of considering the different stages of adolescence and sex when investigating adolescents’ mindfulness and self-compassion and their role in psychological distress.

### The present study

Therefore, this study aims to address three primary objectives. First, the intergenerational transmission mechanisms of psychological distress will be examined. Second, the mediating roles of family emotional climate, mindfulness, and self-compassion will be investigated. Finally, moderating effects of adolescent developmental stage and sex on these pathways will be tested. Specifically, it is hypothesized that:

Hypothesis 1: Family emotional climate mediates the relation-ship between parental and adolescent psychological distress.

Hypothesis 2: Mindfulness and self-compassion in adolescents mediate the relationship between family emotional climate and adolescent psychological distress.

Hypothesis 3: Adolescent developmental stage and sex serve as moderators in the relationships between parents’ psychological distress and the various variables.

## Materials and methods

### Participants and procedure

Three hundred and forty-eight adolescents and their parents participated in this study. Participants were recruited through local schools in Shanxi Province, China. Following ethics approval from the Chinese University, questionnaires were administered to school students during regular class hours. Parent questionnaire was taken home to complete and returned to researchers via teachers. In each family, only one parent completed the survey (mother: 69%; father: 31%). This single-reporter design avoids dyadic interdependence issues as: No families had dual-parent responses and parental responses represent independent observations. The parents’ responses was treated as family-level variable. Prior to the study, written informed consent was obtained from both parents and adolescents, with permission secured through the school principal and participating teachers. Students completed the surveys voluntarily in their classrooms under teacher supervision, and all instruments were subsequently returned to the research team. Eligible participants were school students aged 12–18 years enrolled in Shanxi Province. Participants with a history of severe mental health disorders, cognitive impairments that would hinder participation, or inability to communicate in the study language were excluded. The average age of the adolescent was 14.21 (SD = 1.74) with 57.5% female. The majority (*n* = 205, 58.9%) were in the early stage of adolescence (ages 12–14), 95 (27.3%) were in the middle stage of adolescence (ages 15–16), and 48 (13.8%) were in the late stage of adolescence (ages 17–18). Additional demographic information for the sample is available in Table 1. Adolescents’ age, sex, and grade was collected directly from the adolescents, while data on parental age, sex were reported by parents.

**TABLE 1:** Basic information.

Sample demographics (*N* = 348)		
Variables	Frequency	Percent
**Child sex**		
Girls	200	57.50%
Boys	148	42.50%
**The stage of adolescence**		
Ages 12 to 14	205	58.90%
Ages 15 to 16	95	27.30%
Ages 17 to 18	48	13.80%
**Mothers’ education levels**		
Primary school degree and below	3	0.90%
Secondary school degree	25	7.20%
High school degree	147	42.20%
Junior college	124	35.60%
B.S. degree	29	8.30%
M.S. degree or above	20	5.70%
**Fathers’ education levels**		
Primary school degree and below	5	1.40%
Secondary school degree	24	6.90%
High school degree	136	39.10%
Junior college	124	35.60%
B.S. degree	27	7.80%
M.S. degree or above	32	9.20%

### Measures

#### Adolescents’ mindful attention and awareness

The Mindful Attention Awareness Scale (MAAS; Brown and Ryan, 2003) was used to measure adolescents’ dispositional mindfulness. MAAS is a single factor scale with 15 items using a six-point Likert-type scale (from “almost always” to “almost never”). It has been translated into Chinese and verified in Chinese adolescents (Black et al., 2012). The Cronbach’s alpha for the MAAS in the present study was 0.794.

#### Adolescents’ self-compassion

The Self-Compassion Scale (SCS; Neff, 2003) is a 12-item measure, which assesses three different aspects of self-compassion: Self-kindness (e.g., “I will try to understand and be patient with the traits of my personality that I don’t like.”); Common humanity (e.g., “When I feel lost, I feel that most people are happier than me.”); Mindfulness (e.g., “When I go through tough times, I give myself the care and love that I need.”). Participants were asked to respond on a five-point scale from “almost never” to “almost always.” The Cronbach’s alpha for the SCS in the present study was 0.681.

#### Family emotional climate

The subscales of acceptance and conflict of Family Climate Inventory (Kurdek et al., 1995; Kurdek and Fine, 1993) were used to assess the family emotional climate. Participants were asked to respond on a five-point scale from “strongly disagree” to “strongly agree.” The subscales of the Family Climate Inventory have been used in many previous studies with a good reliability from 0.7 to 0.9 (e.g., Lai and McBride-Chang, 2001). The total score of acceptance subscale and conflict subscale were used to assess the family emotional climate in the present study. The Cronbach’s alpha for the present sample was 0.780.

#### Depression anxiety stress

Depression Anxiety Stress Scales (DASS; Lovibond and Lovibond, 1995) are a 21-item measure consisting of three subscales: depression, anxiety, and stress. Items were scored on a four-point scale ranging from “almost never” to “almost always.” Previous studies have applied DASS to assess the general psychological distress in the sample of adolescents (Wang et al., 2011). In the present study, Cronbach’s alphas for the parental were 0.936, and the subscales of DASS-21 for the parental were 0.88 (depression), 0.85 (anxiety), 0.78 (stress). Cronbach’s alpha for the adolescent was.892, and the subscales of DASS-21 for the adolescent were 0.82 (depression), 0.75 (anxiety), and 0.70 (stress).

### Data analysis

The data analyses were conducted using the SPSS 26.0 and Amos 24.0 in the current study. Descriptive statistics were computed for all sociodemographic and study variables. Then, Pearson correlations between the study variables were computed. To examine whether parents’ psychological distress was associated with adolescents’ psychological distress through family emotional climate, adolescents’ mindfulness and self-compassion, a path model was tested using the maximum likelihood estimation method. The statistical significance of the indirect effects was estimated using bootstrap resampling procedures with 5,000 samples and a 95% bias-corrected confidence interval. The specific indirect effects and the corresponding confidence intervals were estimated using an AMOS user-defined estimate. Regression analysis was used to examine whether adolescent sex and age moderated the relationships between parents’ psychological distress and the various variables.

## Results

### Common variance analysis

Harman’s single factor test was used to exclude common method deviation caused by the questionnaire method. The results showed that there were 21 factors with eigenvalues greater than 1, and the variation explained by the first factor was 14.699% which is less than the critical value of 40%, indicating that the effect of common method deviation would not affect our data result (Podsakoff et al., 2003).

### Descriptive statistics and correlations analyses

The descriptive statistics of all variables, including parents’ psychological distress, adolescents’ mindfulness and self-compassion and adolescents’ psychological distress, are displayed in Table 2. The correlations analyses in the main variables are shown in Table 2. The results indicate that parents’ psychological distress was negatively associated with mother’s education level (*r* = −0.205, *p* < 0.01), father’s education level (*r* = −0.196, *p* < 0.01) and adolescents’ sex (*r* = −0.119, *p* < 0.05). Parents’ psychological distress was negatively correlated with family emotional climate (*r* = −0.108, *p* < 0.05) and adolescents’ mindfulness (*r* = −0.117, *p* < 0.05), and was positively correlated with adolescents’ psychological distress (*r* = 0.107, *p* < 0.05). Family emotional climate was positively correlated with adolescents’ mindfulness (*r* = 0.284, *p* < 0.01) and self-compassion (*r* = 0.185, *p* < 0.01), and was negatively correlated with adolescents’ psychological distress (*r* = −0.323, *p* < 0.01). And adolescents’ mindfulness was positively correlated with adolescents’ self-compassion (*r* = 0.373, *p* < 0.01), and was negatively correlated with adolescents’ psychological distress (*r* = −0.607, *p* < 0.01). Adolescents’ self-compassion was negatively correlated with adolescents’ psychological distress (*r* = −0.474, *p* < 0.01).

**TABLE 2:** Means, standard deviations, and correlations between study variables.

Variables	M	SD	Range	1	2	3	4	5	6	7	8	9
1. Sex	–	–	–	–	–	–	–	–	–	–	–	–
2. Age	14.10	2.03	12–18	0.083	–	–	–	–	–	–	–	–
3. Mother’s education level	2.61	0.97	–	0.039	0.038	–	–	–	–	–	–	–
4. Father’s education level	2.69	1.07	–	0.034	0.076	0.628**	–	–	–	–	–	–
5. Parents’ DASS	16.38	11.0	0.00–61.00	−0.119*	0.061	−0.205**	−0.196**	–	–	–	–	–
6. Family emotional climate	46.62	7.35	20.00–60.00	0.020	−0.071	0.054	−0.028	−0.108*	–	–	–	–
7. Mindfulness	59.74	12.15	25.00–90.00	0.021	−0.099	−0.006	−0.030	−0.117*	0.284**	–	–	–
8. Self-compassion	37.05	6.14	17.00–54.00	−0.049	−0.039	0.016	−0.040	−0.085	0.195**	0.373**	–	–
9. Adolescents’ DASS	20.95	10.66	0.00–59.00	0.024	0.072	−0.048	−0.008	0.107*	−0.323**	−0.607**	−0.474**	–

DASS, depression anxiety stress; *, *P* < 0.05; **, *P* < 0.01; ***, *P* < 0.001.

### Direct and indirect effects analysis

**FIGURE 1:**
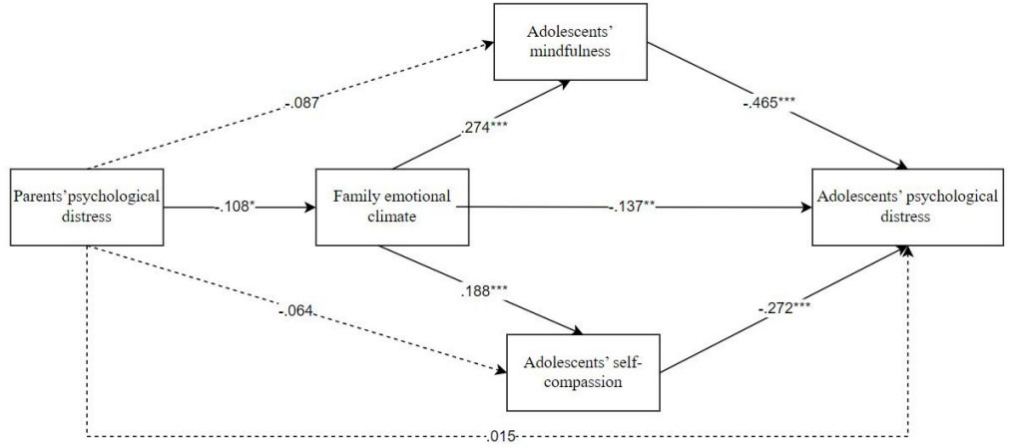
Path model examining the associations between parents’ psychological distress and adolescents’ psychological distress through family emotional climate, adolescents’ self-compassion and mindfulness. Path values represent standardized regression coefficients. For simplicity, covariates and measurement error terms are not shown, *p < 0.05, **p < 0.01, ***p < 0.001.

For each path in the model (Figure 1), parents’ psychological distress negatively predicted family emotional climate (β = −0.108, *p* < 0.05). Family emotional climate positively predicted adolescents’ mindfulness (β = 0.274, *p* < 0.001) and self-compassion (β = 0.188, *p* < 0.001), but negatively predicted adolescents’ psychological distress (β = −0.137, *p* < 0.01). Adolescents’ mindfulness (β = −0.465, *p* < 0.001) and self-compassion (β = −0.272, *p* < 0.001) negatively predicted adolescents’ psychological distress. However, parents’ psychological distress did not significantly predict adolescents’ mindfulness (β = −0.087, *p* > 0.05), adolescents’ self-compassion (β = −0.064, *p* > 0.05) and adolescents’ psychological distress (β = 0.015, *p* > 0.05).

With regard to the specific indirect effects (Table 3), parents’ psychological distress had an indirect effect on adolescents’ mindfulness and self-compassion and adolescents’ psychological distress via family emotional climate, and the indirect effect was −0.033, −0.011 and 0.014, 95% CI = (−0.074, −0.003), (−0.029, −0.001), and (0.001, 0.038). In addition, parents’ psychological distress had an indirect effect on adolescents’ psychological distress through family emotional climate and adolescents’ mindfulness, the indirect effect was 0.013, 95% CI = (0.001, 0.031). Parents’ psychological distress had an indirect effect on adolescents’ psychological distress through family emotional climate and adolescents’ self-compassion, the indirect effect was 0.005, 95% CI = (0.001, 0.015). Finally, family emotional climate had an indirect effect on adolescents’ psychological distress through adolescents’ mindfulness and self-compassion, the indirect effect was −0.185 and −0.074, 95% CI = (−0.270, −0.111) and (−0.134, −0.038).

**TABLE 3:** Direct and indirect effects of parents’ psychological distress on adolescents’ psychological distress.

Indirect effect	Unstandardized coefficients	Standard error	*P*-value	BC95% CI lower/upper
PDASS→FEC→Mindfulness	−0.033	0.018	0.033	−0.074/−0.003
PDASS→FEC→Self-compassion	−0.011	0.007	0.024	−0.029/−0.001
PDASS→FEC→ADASS	0.014	0.009	0.029	0.001/0.038
PDASS→Mindfulness→ADASS	0.039	0.023	0.072	−0.004/0.086
PDASS→Self-compassion→ADASS	0.014	0.014	0.181	−0.009/0.045
PDASS→FEC→Mindfulnesss→ADASS	0.013	0.007	0.032	0.001/0.031
PDASS→FEC→Self-compassion→ADASS	0.005	0.003	0.019	0.001/0.015
FEC→Mindfulnesss→ADASS	−0.185	0.040	0.000	−0.270/−0.111
FEC→Self-compassion→ADASS	−0.074	0.026	0.000	−0.134/−0.031

PDASS, parents’ psychological distress; ADASS, adolescents’ psychological distress; FEC, family emotional climate.

### Test for moderation effects

To test whether sex and age have moderating effects, this study ran regression analyses, with the results presented in Table 4. It is worth noting that although three developmental stages were considered (early adolescence - 12–14 years; middle adolescence - 15–16 years; and late adolescence - 17–18 years) (Spano, 2004), due to the small number of adolescents in late adolescence in this study, middle and late adolescence were combined into one category. The findings indicate that sex and age do not function as moderators in the model.

**TABLE 4:** Test for moderation effects.

Variables	Mindfulness		Mindfulness		Self-compassion		Self-compassion		FEC		FEC		ADASS		ADASS	
	β	*t*	β	*t*	β	*t*	β	*t*	β	*t*	β	*t*	β	*t*	β	*t*
PDASS	−0.044	−0.804	0.237	1.418	−0.089	−1.646	−0.335	−2.003	−0.107	−1.987	−0.113	−0.674	0.108	2.009	0.003	0.020
Sex	−0.025	−0.462	−0.024	−0.447	−0.033	−0.603	−0.033	−0.619	0.01	0.193	0.01	0.192	0.014	0.251	0.013	0.245
Int			−0.297	−1.773			0.259	1.554			0.006	0.035			0.111	0.663
*R* ^2^	0.002		0.011		0.008		0.015		0.012		0.012		0.012		0.013	
*F*	0.390		1.310		1.436		1.767		2.072		1.378		2.018		1.489	
PDASS	−0.101	−1.918	−0.027	−0.168	−0.102	−1.904	−0.085	−0.523	−0.083	−1.536	−0.097	−0.592	0.096	1.796	−0.028	−0.175
Age	−0.176	−3.322	−0.175	−3.301	−0.074	−1.386	−0.074	−1.38	−0.026	−0.475	−0.026	−0.477	0.126	2.368	0.125	2.339
Int			−0.079	−0.491			−0.018	−0.11			0.015	0.091			0.131	0.812
*R* ^2^	0.044		0.045		0.017		0.017		0.008		0.008		0.027		0.029	
*F*	7.969		5.381		3.025		2.015		1.366		0.911		4.82		3.43	

## Discussion

This study advances understanding of the mechanisms linking parental and adolescent psychological distress by establishing family emotional climate, mindfulness, and self-compassion as critical mediators. These findings align with existing evidence demonstrating that parental psychological distress predicts adolescent mental health outcomes (Sawyer et al., 2019), and that mindfulness and self-compassion correlate positively with psychological well-being while inversely associating with negative affect (Jimenez et al., 2010). Crucially, we extend prior research showing negative family emotional climates associate with adolescent depression and anxiety by demonstrating how such climates transmit distress specifically through compromised adolescent mindfulness and self-compassion.

As expected, parental psychological distress was associated with adolescent psychological distress. This aligns with Nomura et al. (2007), who found that children of depressed parents face elevated depression risk. Further supporting this link, many adolescents indirectly experience parental trauma or imagine awareness of their parents’ traumatic events (Bowers and Yehuda, 2016). Such exposure may cause adolescents to feel stressed and withdraw from developmentally beneficial parent-child interactions, ultimately reducing their social competence and self-efficacy while increasing vulnerability to psychological distress (Sun et al., 2022). However, our results revealed that while parental distress correlates with adolescent distress, it does not directly predict it. This finding echoes Eley et al. (2015), who demonstrated that genetic factors play a limited role in anxiety transmission, suggesting mediation through family processes. Related studies have also found mediators or moderators of intergenerational transmission between parental and adolescent psychological distress (e.g., co-parenting, parent-child attachment, environment) (Liu et al., 2021). Crucially, our identification of full mediation indicates that parental distress first permeated family systems and individual psychological resources before manifesting in adolescent outcomes. As conceptualized by Bögels and Brechman-Toussaint (2006), parental negative emotions represent the absence of warmth and acceptance within the family unit. This situation can negatively impact parenting behaviors and the developmental environment that parents foster, thereby promoting the emergence and escalation of adolescent anxiety (Bögels and Brechman-Toussaint, 2006). The absence of direct effects demonstrates that parental distress influences adolescents exclusively through disruption of emotional climate and psychological ecosystems; it further confirms that intergenerational transmission is neither inevitable nor deterministic. Consequently, effective interventions must target multi-systemic pathways—addressing both family-level processes (emotional climate) and individual-level psychological resources (mindfulness, self-compassion).

This study confirms that the link between parental and adolescent psychological distress is indirectly mediated by the family emotional climate, mindfulness, and self-compassion in adolescents. Parental psychological distress compromises adolescents’ psychological distress by altering the family emotional climate, which echoes previous research findings (Eley et al., 2015). Prolonged parental anxiety, depression, or other forms of psychological distress can create a tense and unstable family atmosphere. Adolescents raised in such environments are more prone to experiencing psychological distress (Neff and McGehee, 2010). This mechanism aligns with Hammen and Brennan’s (2002) community-based study of 800 mother-adolescent dyads, which established that maternal depression predicts youth depression through stress generation in family environments. Their results empirically substantiate our model: stressful family climates mediate the transmission of psychological distress across generations.

Our findings indicate that adolescents from supportive family environments tend to exhibit higher mindfulness and self-compassion than those from problematic family settings. This is likely because individuals often internalize emotional patterns and processing styles from their family environment (Stuart and Jose, 2012). A positive family climate offers timely external resources like parental support and effective coping strategies. This enables adolescents to enhance self-regulation, reduce impulsive reactions, and boost mindfulness. Prior research also suggests that a positive family environment may positively influence a person’s internal compassionate dialog. Conversely, dysfunctional family environments are linked to self-criticism and a lack of self-compassion (Neff and McGehee, 2010). In warm, low-conflict families, adolescents receive more support, attention, and empathy, fostering secure attachment crucial for developing mindfulness and self-compassion. In contrast, tense family environments can hinder the parent-adolescent bond, making it difficult for adolescents to develop compassion and leading to stress hypersensitivity (Kraaij et al., 2003). In summary, children from positive family environments internalize a positive, self-compassionate dialog, while those from negative environments may internalize negative thought patterns (Moreira et al., 2018). Thus, family emotional climate plays a key role in shaping adolescents’ psychological processes as a vital social support source.

The results showed that mindfulness and self-compassion were negatively correlated with adolescents’ psychological distress. This is consistent with Makadi and Koszycki (2019), who found that higher levels of self-compassion and mindfulness are linked to lower stress, anxiety, and depression, as well as greater life satisfaction and better psychological functioning. Mindfulness helps individuals monitor their emotions and related thoughts, approach suffering with non-judgment and acceptance, and reduce automatic reactions (Gross, 2014). Concurrently, self-compassion enhances the ability to transform self-criticism into a positive self-view and fosters a caring attitude toward oneself, leading to psychological benefits and reducing the development of psychological distress in adolescents (Neff, 2003; Zhou et al., 2022). Together, these mechanisms explain how mindfulness and self-compassion buffer against adolescents’ psychological distress.

The absence of sex and age moderation effects may be explained through adolescence’s status as a sensitive period for socioemotional learning, wherein environmental influences substantially outweigh innate differences (Fuhrmann et al., 2015). Heightened neuroplasticity during this developmental phase renders adolescents receptive to familial cues—especially parental psychological states and emotional expressions—which likely attenuates demographic variations. Although the cross-sectional design of this study does not allow us to draw conclusions about causality, these findings suggest that the role of family emotional environment, self-compassion, and mindfulness on adolescent psychological distress may be equally relevant and strong at all stages of adolescence and between boys and girls. Future longitudinal studies are necessary to confirm the variability in pathway models by stage and sex in adolescence.

## Limitations, future directions, and implications

When generalizing the results of this study, several limitations should be considered. First, despite being grounded in existing theory and literature, the cross-sectional design of this study precludes the establishment of causal relationships between variables. Future research should employ a longitudinal design to better confirm the causal links between the variables examined. Second, the study relied solely on self-report measures from parents and adolescents. This raises questions about whether these reports accurately reflect family dynamics and individual states or if they might instead represent a negative response bias related to depressive symptoms. To enhance the validity of the findings, future studies should incorporate a variety of measurement methods and sources, such as behavioral assessments and other non-self-report measures. And we acknowledge the inherent dyadic non-independence between parent-adolescent pairs was not statistically accounted for. Although traditional SEM assumes observation independence, familial relationships naturally violate this assumption. Future research should implement actor-partner interdependence models (APIM) or multilevel frameworks to disentangle these effects. Third, the internal consistency of the Self-Compassion Scale (SCS) in this study was suboptimal, though within the acceptable range for exploratory research. Lower reliability could attenuate observed effect sizes, potentially obscuring the true strength of associations involving self-compassion. Future research should consider employing alternative or additional measures of self-compassion with established higher reliability to better capture this construct. Fourth, the sample size constrains statistical power to detect smaller effects and limits multi-group analyses. Additionally, We only selected data from some schools in a specific region. Adolescents’ socio-economic status and psychological profiles may systematically differ across educational contexts (e.g., public vs. private schools, urban vs. rural districts). Future research should recruit larger, more demographically diverse cohorts to validate these findings. Lastly, this study primarily examined how family emotional climate negatively impacts mindfulness and self - compassion, and thereby affects psychological distress. Future research should provide more empirical evidence on the positive effects of external family context and internal factors like mindfulness and self - compassion on adolescents’ emotion regulation and positive emotions. This will help develop a more comprehensive understanding of emotion regulation.

This study offers valuable insights into adolescent emotion regulation from both theoretical and practical angles. Theoretically, prior studies have proposed that both internal and external factors can influence emotion regulation in individuals. However, few have empirically examined how these factors interact in adolescents. This study bridges this gap by simultaneously exploring the impacts of internal factors (mindfulness and self - compassion) and an external factor (family emotional climate) on adolescents’ emotion regulation and psychological distress. The findings extend our understanding of the interplay between these elements in the context of adolescent emotion regulation research. Practically, this study suggests that effective prevention programs should be implemented to enhance adolescents’ mindfulness, self-compassion, and foster a positive family climate. Studies have shown that mindfulness-based programs can boost adolescents’ well-being and emotion regulation abilities (e.g., Broderick and Metz, 2009). Additionally, mindful parenting programs have been confirmed to improve parents’ emotion regulation and well-being, leading to positive parent-child relationships and better outcomes for children (Bögels et al., 2014). Thus, mindfulness-based programs for both adolescents and parents show great promise in reducing adolescents’ psychological distress by enhancing their mindfulness, self-compassion, and family environment.

## Data Availability

The raw data supporting the conclusions of this article will be made available by the authors, without undue reservation.
